# PLEKHO2 inhibits TNFα-induced cell death by suppressing RIPK1 activation

**DOI:** 10.1038/s41419-021-04001-2

**Published:** 2021-07-16

**Authors:** Chenchen Zhou, Xueli Zhang, Cuiping Yang, Yuan He, Luo Zhang

**Affiliations:** 1 Department of Biomedical Engineering, the Fifth medical Centre, Chinese PLA General Hospital, Beijing, 100071 China; 2 Department of pathology, the Fifth medical Centre, Chinese PLA General Hospital, Beijing, 100071 China; 3 Department of Respiratory and Critical Care Medicine, the Fifth medical Centre, Chinese PLA General Hospital, Beijing, 100071 China; 4 Research Center of Bioengineering, the Medical Innovation Research Division of Chinese PLA General Hospital, Beijing, 100039 China

**Keywords:** Apoptosis, Ubiquitylation

## Abstract

Receptor interaction protein kinase 1 (RIPK1) plays a diverse role in tumor necrosis factor α (TNFα) signalings. The ubiquitination of RIPK1 is essential for NF-κB activation, whereas its kinase activity promotes apoptosis and necroptosis. However, the mechanisms underlying have not been fully illuminated. Here we report that PH domain-containing family O member 2 (PLEKHO2) inhibits RIPK1-dependent cell death and is necessary for NF-κB activation in response to TNFα. Cells of PLKEHO2 deficiency are more susceptible to TNF-α induced apoptosis and necroptosis with increased RIPK1 activation, which is consistent with the observation that the susceptibility of PLEKHO2−/− cells is effectively prevented by treatment of RIPK1 kinase inhibitor. Moreover, PLEKHO2 deficient cells exhibit compromised RIPK1 ubiquitination and NF-κB activation in response to TNFα. Ultimately, PLEKHO2-deficient mice display greatly increased hepatotoxicity and lethality after TNFα-induced hepatitis. In summary, our study revealed that PLEKHO2 is a novel inhibitor of apoptosis and necroptosis, which plays a key role in regulating RIPK1 ubiquitination and activation

## Introduction

Tumor necrosis factor α (TNFα) is a pro-inflammatory cytokine that has important roles in immunity and cellular homeostasis [1]. In most cells, TNFα stimulation is not cytotoxic and induces direct pro-inflammatory signaling. Binding to TNFR1, TNFα induces the formation of complex I, comprising TNFR1-associated death domain protein (TRADD) [2], receptor interaction protein kinase 1(RIPK1) [3] and the E3 ubiquitin ligases TNF-receptor-associated factor 2 (TRAF2) [4], the cellular inhibitors of apoptosis (cIAP1 or cIAP2) [5] and the linear ubiquitin chain assembly complex (LUBAC) [6, 7]. TRADD is required for the recruitment of TRAF2, cIAP1/2, and LUBAC, and for the ubiquitylation of RIPK1 with K63 and linear chains within complex I [8, 9]. This complex favors proinflammatory signaling and prevents cell death by leading to NF-κB and mitogen-activated protein kinase (MAPK) activation, which induce antiapoptotic genes such as cellular FLICE inhibitory protein (c-FLIP) and cIAPs. Destabilization of complex I lead to the formation of a second cytosolic complex IIa, consisting of RIPK1, FADD, and caspase-8 [10]. The protein synthesis inhibitor cycloheximide (CHX) renders cells sensitive for this complex IIa-mediated apoptosis [11]. In conditions such as TNFα stimulation in the presence of IAP inhibitors (Smac mimetics) or knockdown of IAPs [11], TAK1 inhibition or knockdown [12], a cytosolic complex IIb forms that is composed of RIPK1, RIPK3, FADD, and caspase-8. This complex IIb favors RIPK1-kinase-activity-dependent apoptosis. However, the mechanisms underlying this process have not been fully illuminated.

Pleckstrin homology (PH)-domain-containing family O member 2(PLEKHO2; also known as PLEKHO2), is a member of the PH-domain-containing protein superfamily. The PH domain consists of a ~100-amino acid region that occurs twice in pleckstrin and in numerous other proteins involved in cell signaling. Although it was originally proposed that PH domains were involved in protein–protein interactions, subsequent reports indicated that many PH domains participate in cell membrane targeting by binding to phosphoinositides [13]. The human genome contains ~350 PH domain proteins, however, few of these have had their functions convincingly demonstrated. We previously reported that PLEKHO2 plays an impotent role in macrophage survival. PLEKHO2-deficient mice exhibit an apparent reduction in the macrophage population, PLEKHO2 deficiency in macrophage results in increased apoptotic cell death and elevated activity of caspase-3 [14].

We now demonstrate that PLEKHO2 plays a critical role in preventing TNFα-induced cell death. Genetic deficiency of PLEKHO2 sensitizes cells to TNFα plus CHX or SAMC induced apoptosis and necroptosis. The excessive cell death in PLEKHO2−/− cells can be effectively prevented by Nec-1 treatment is proved to be dependent on the kinase activity of RIPK1. Furthermore, compromised RIPK1 ubiquitination and NF-κB activation were observed in PLEKHO2−/− cells. Moreover, PLEKHO2-deficient mice display greatly increased hepatotoxicity and ultimately lethality after TNFα-induced hepatitis. Thus, PLEKHO2 is a novel regulator of cell death in response to TNFα.

## Results

### PLEKHO2 deficiency sensitizes cells to TNFα and CHX induced apoptosis

**Fig. 1:**
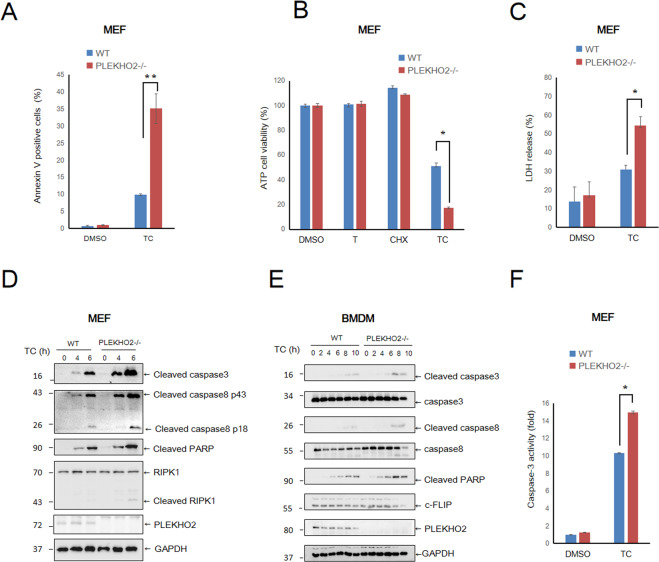
PLEKHO2 deficiency sensitizes cells to TNFα/CHX induced apoptosis. **A** WT and PLKEHO2−/− MEFs were treated with DMSO or murine TNFα (20 ng/mL) and cycloheximide (CHX) (10 μg/mL) for 8 h and analyzed by flow cytometry for a percentage of cells staining positive for annexin V staining. **B** WT and PLKEHO2−/− MEFs treated as indicated (TC, TNFα 20 ng/mL plus CHX 10 μg/mL) for 8 h, cell viability was measured by ATP level. **C** WT and PLKEHO2−/− MEFs treated with DMSO or TNFα plus CHX (TC) as **B** for 8 h. Cytotoxicity was measured by LDH release. **D** WT and PLKEHO2−/− MEFs were treated with TNFα (20 ng/mL) and cycloheximide (CHX) (10 μg/mL) for indicated periods of time. Cell lysates were probed with indicated antibodies. **E** WT and PLKEHO2−/− BMDMs were treated with TNFα (20 ng/mL) and cycloheximide (CHX) (10 μg/mL) for indicated periods of time. Cell lysates were probed with indicated antibodies. **F** WT and PLKEHO2−/− MEFs treated with DMSO or TNFα plus CHX (TC) for 8 h, Caspase-3 activity was measured by Caspase-Glo 3 assay. Results are representative of at least three independent experiments.

In order to define the role of PLEKHO2 in TNF-α signaling, PLEKHO2-deficient mice were used. Mouse embryonic fibroblasts (MEFs) derived from wild-type (WT) and PLEKHO2−/− littermate was cultured to assess the effects of PLEKHO2 deficiency on apoptosis. The expression of PLEKHO2 in MEFs by western blot is shown in Fig. S1A. Compared to DMSO treatment, co-stimulation of MEFs with TNFα and CHX caused the cells to become rounded and non-adherent but the number of such cells was greatly increased when PLEKHO2 was deficient (Fig. S1B). Flow cytometry analyses indicated that Annexin V positive cells increased after TNFα/CHX (TC) treatment, but such changes were much more apparent in PLEKHO2−/− MEFs relative to their WT control cells (Fig. 1A and Fig. S1C). Furthermore, we observed that the cell viability of PLEKHO2−/− MEFs measured by ATP level dropped significantly after treatment with TNF-α/CHX compared with that of WT MEFs (Fig. 1B). Meanwhile, LDH released cell death assay also indicated that PLEKHO2−/− MEFs were susceptible to TC-induced cell death (Fig. 1C).

Next, we characterized the levels of key proteins involved in regulating TNFα-mediated apoptosis in MEFs and bone marrow-derived macrophages (BMDMs). The levels of cleavage of caspase-8, caspase-3, and PARP-1, important hallmarks of apoptosis induced by TC, were elevated in PLEKHO2−/− cells compared with that of WT (Fig. 1D, E), consistent with increased sensitivity of PLEKHO2−/− cells to apoptosis. Additionally, reconstitution of WT PLEKHO2 in PLEKHO2−/− MEF diminished the protein levels of apoptotic hallmarks (Fig. S1D) but knockdown of PLEKHO2 by shRNA in RAW264.7 macrophage cells enhanced the levels of these hallmarks (Fig. S1E) and sensitized cell death (Fig. S1F). These changes were associated with greater activity of caspase-3, an executor of apoptosis, in PLEKHO2−/− MEFs as well (Fig. 1F). Furthermore, we noticed that PLEKHO2 deficiency resulted in decreased c-FLIP level (Fig. 1E and Fig. S1D) accompanied by caspase-8 activation. Taken together, these data suggest that PLEKHO2 deficiency sensitizes cells to apoptosis in response to TNFα and CHX.

### PLEKHO2 deficiency sensitizes cells to TNFα and Smac mimetic induced apoptosis

**Fig. 2:**
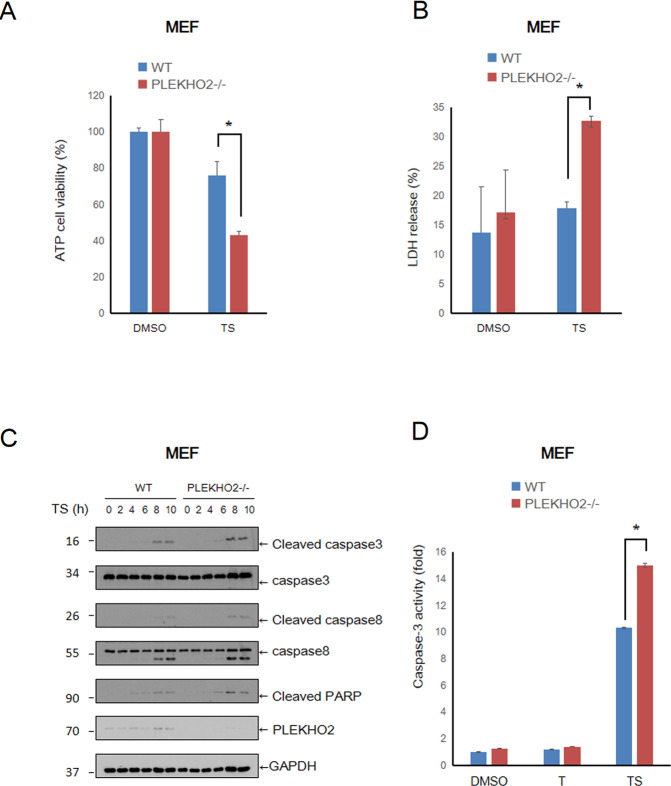
PLEKHO2 deficiency sensitizes cells to TNFα/Smac mimetic induced apoptosis. **A** WT and PLKEHO2−/− MEFs were treated with DMSO or TS (TNFα 20 ng/mL plus Smac mimetic 100 nM) for 8 h, cell viability was measured by ATP level. **B** WT and PLKEHO2−/− MEFs treated with DMSO or TS as **A** for 8 h. Cytotoxicity was measured by LDH release. **C** WT and PLKEHO2−/− MEFs were treated with TS as **A** for indicated periods of time. Cell lysates were probed with indicated antibodies. **D** WT and PLKEHO2−/− MEFs treated with DMSO or TS as **A** for 8 h, Caspase-3 activity was measured by Caspase-Glo 3 assay. Results are representative of at least three independent experiments.

We then investigated the effect of PLEKHO2 in TNFα and Smac mimetic induced apoptosis. Compared to DMSO treatment, co-stimulation with TNFα and Smac mimetic (TS) caused decreased cell viability of PLEKHO2−/− MEFs measured by ATP level compared with that of WT MEFs (Fig. 2A). Meanwhile, LDH released cell death assay also indicated that PLEKHO2−/− MEFs were susceptible to TS-induced cell death (Fig. 2B). Furthermore, the levels of cleavage of caspase-8, caspase-3, and PARP-1, important hallmarks of apoptosis induced by TS, were elevated in PLEKHO2−/− cells compared with that of WT (Fig. 2C). Caspases-3 activity assay also indicated an inhibiting role of PLEKHO2 in TS induced Caspases-3 activation (Fig. 2D). These data together suggest that PLEKHO2 deficiency results in sensitization to TNFα and Smac mimetic-induced apoptosis.

### PLEKHO2 deficiency facilitates necroptosis and activation of RIPK1

**Fig. 3:**
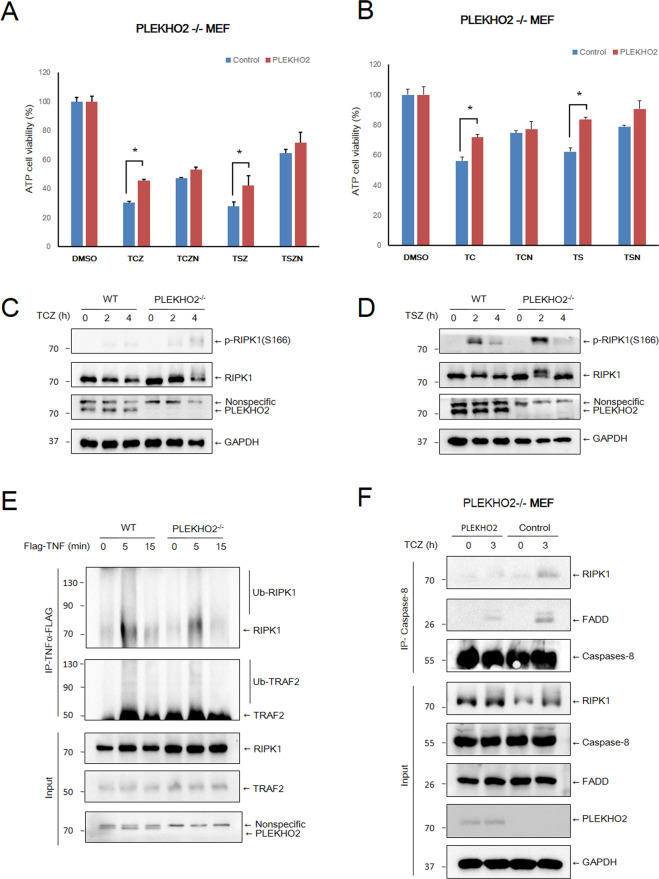
PLEKHO2 deficiency facilitates necroptosis and activation of RIPK1. **A** Control and PLEKHO2 reexpressed PLKEHO2−/− MEFs were treated with DMSO, TCZ, TCZN, TSZ, and TSZN for 8 h, cell viability was measured by ATP level. Abbreviations are as follows: TSZ TNF-α (20 ng/ml) + Smac mimetic (100 nM) + zVAD (20 μM), TSZN TNF-α + Smac mimetic + zVAD + Necrostatin-1 (20 μM), TCZ TNF-α (20 ng/ml) + CHX (10 μg/mL) + zVAD (20 μM), TCZN TNF-α + CHX + zVAD + Necrostatin-1 (20 μM). **B** Control and PLEKHO2 reexpressed PLKEHO2−/− MEFs treated with DMSO, TS, TSN, TC, and TCN for 8 h, cell viability was measured by ATP level. Abbreviations are as follows: TS TNF-α (20 ng/ml) + Smac mimetic (100 nM), TSN TNF-α + Smac mimetic + Necrostatin-1 (20 μM), TC TNF-α + CHX (20 ng/ml), TCN TNF-α + CHX + Necrostatin-1 (20 μM). **C** WT and PLKEHO2−/− MEFs were treated with TCZ, TCZ, TNF-α (20 ng/ml) + CHX (10 μg/mL) + zVAD (20 μM) for indicated periods of time. Cell lysates were probed with indicated antibodies. **D** WT and PLKEHO2−/− MEFs treated with TSZ, TNF-α (20 ng/ml) + Smac mimetic (100 nM) + zVAD (20 μM) for indicated periods of time. Cell lysates were probed with indicated antibodies. **E** WT and PLKEHO2−/− MEFs were treated with Flag-TNF-α (100 ng/ml) for the indicated periods of time, complex I was immunoprecipitated using anti-FLAG beads, endogenous RIPK1 ubiquitination, and indicated proteins were detected by western blotting. **F** Control and PLEKHO2 reexpressed PLKEHO2−/− MEFs were treated with TCZ, TCZ, TNF-α (20 ng/ml) + CHX (10 μg/mL) + zVAD (20 μM) for 3 h, and then Caspase-8 was immunoprecipitated (IP). IP and cell lysates were analyzed by western blotting using the indicated antibodies. Results are representative of at least three independent experiments.

We next asked whether PLEKHO2 played a role in TNFα induced necroptosis. Using PLEKHO2−/− MEFs complemented with WT PLEKHO2, we found that the stimulation of TNFα/CHX/zVAD (TCZ) or TNFα/Smac/zVAD (TSZ) induced a higher level of cell death in control PLEKHO2−/− MEFs than complemented ones. Meanwhile, treatment of Necrostatin-1(Nec-1 or N), a RIPK1 kinase inhibitor, efficiently prolonged the survival of PLEKHO2−/− MEFs in response to TCZ or TSZ induced necroptosis (Fig. 3A). As Nec-1 also blocks apoptosis induced by TS or TC in some cases, we then investigated whether Nec-1 rescue PLEKHO2−/− MEFs in response to TC and TS. We indeed found Nec-1 treatment enhanced cell survival and almost diminished the difference between control and complemented PLEKHO2−/− MEFs (Fig. 3B). Consistently, we found that the level of p-Ser166 RIPK1, a marker of RIPK1 activation, was elevated in PLEKHO2−/− MEFs than that of WT MEFs both in response to TCZ and TSZ (Fig. 3C, D). As the ubiquitination of RIPK1 in complex I prevents its activation, we next investigated whether PLEKHO2 effect this process. Within 5 min of TNFα stimulation, obvious ubiquitination of RIPK1 in complex I was detected in WT cells, whereas a reduction in RIPK1 and TRAF2 ubiquitination was observed in PLEKHO2−/− MEFs (Fig. 3E). It has been shown that the activation of RIPK1 kinase activity is critical for the transition from complex I to complex II, we therefore, tested whether PLEKHO2 affects the formation of complex II. As expected, a greater increased level of RIPK1 and FADD interaction with caspase 8 was found in control PLEKHO2−/− MEFs than that of PLEKHO2 complemented cells after TCZ treatment (Fig. 3F). Therefore, we conclude that PLEKHO2 deficiency facilitates necroptosis and activation of RIPK1.

### PLEKHO2 deficiency results in impaired NF-κB activation in response to TNFα

**Fig. 4:**
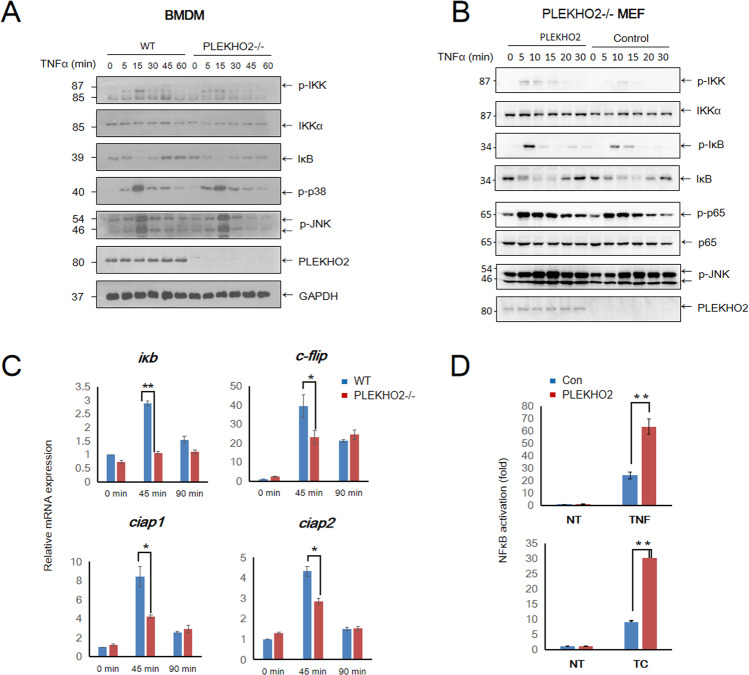
PLEKHO2 promotes TNFα-induced NF-κB activation. **A**, **B** WT and PLKEHO2−/− BMDMs (**A**) or Control and PLEKHO2 reexpressed PLKEHO2−/− MEFs (**B**) were treated with murine TNFα (20 ng/mL) for indicated periods of time, and cell lysates were probed with indicated antibodies. **C** mRNA expression of a panel of NF-κB targeted genes in WT or PLKEHO2−/− MEFs treated with TNFα (20 ng/mL) for indicated periods of time were determined by quantitative PCR (qPCR). **D** Reporter assay analysis of NF-κB activation in 293T cells expressing PLEKHO2 or not, treated with TNFα (20 ng/mL) or TNFα plus CHX. Results are representative of at least three independent experiments.

To determine if the impaired ubiquitination of RIPK1 in PLEKHO2−/− cells effects NF-κB activation, we performed a time-course experiment by treating cells with TNF-α. As expected, the phosphorylation of IKK in response to TNFα stimulation was weaker and less sustained in PLEKHO2−/− BMDMs and this was consistent with decreased IκB reexpression after its degradation. However, there were no obvious differences of JNK and p38 phosphorylation between WT and PLEKHO2−/− BMDMs (Fig. 4A) and a similar result was obtained in MEFs (Fig. 4B). Furthermore, the TNFα-induced expression of mRNA levels of the NF-κB-responsive genes c-FLIP, IκB, cIAP1, and cIAP2 reduced significantly in PLEKHO2−/− MEFs (Fig. 4C). In additional experiments, we examined the influence of PLEKHO2 expression on TNFα induced NF-κB activation in HEK 293 T using an NF-κB promoter luciferase-based reporter system. We found that activation of NF-κB was augmented by expression of PLEKHO2 after treatment with TNFα or TC (Fig. 4D). Thus, these data suggest a key role of PLEKHO2 in regulating NF-κB signaling in response to TNFα.

### PLEKHO2 deficiency in mice leads to increased hepatoxicity and lethality in response to TNFα

**Fig. 5:**
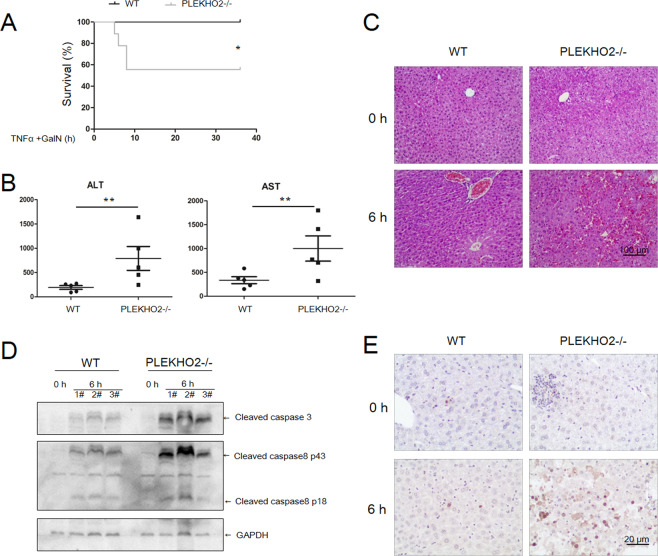
PLEKHO2 deficiency in mice leads to increased hepatoxicity and lethality in response to TNFα in vivo. **A** Age- and sex-matched WT and PLEKHO2−/− mice were injected intraperitoneally with TNFα (2.5 ng/g body weight) plus d-galactosamine (0.8 μg/g body weight). Survival was monitored every hour for 36 h (*p < 0.05, one-way ANOVA). **B** Mice were treated as **A**, serum AST and ALT enzyme levels were measured 6 h after TNFα/d-GalN injection. **C**, **E** Mice were treated as **A**, 6 h after, hepatic tissue was collected, sectioned, and subjected to histological analysis using H&E (**C**), WB analysis (**D**), and TUNEL staining (**E**).

The findings above provide strong evidence for the role of PLEKHO2 in regulating apoptosis in response to TNFα in vitro. Next, we investigated the in vivo relevance of this role using PLEKHO2-deficient mice. To do this, the well-established liver injury model was employed in which the hepatotoxic agent d-galactosamine (d-GalN) sensitizes mice to the lethal effects of low doses of TNFα15. We co-administered d-GalN with TNFα that failed to render WT mice sensitive to the lethal effects (*n* = 8). However, under these same conditions, only 44.4% PLKEHO2−/− mice (*n* = 9) survived at 36 h after treatment (Fig. 5A). Given that hepatoxicity is the key contributing factor to the lethal effects in this model, serum ALT and AST levels as well as liver tissue were examined from the different mouse cohorts. Compared with WT mice, the ALT and AST levels of PLEKHO2−/− mice markedly elevated at 6 h after TNFα/d-GalN treatment (Fig. 5B). We also observed more multifocal areas of hemorrhage in the liver of PLEKHO2−/− mice than that of WT (Fig. 5C). Consistent with these observations, immunoblot analysis of liver extracts from TNFα/d-GalN and control animals revealed extensive processing of caspase-3 and caspase-8 in PLEKHO2−/− mice (Fig. 5D). Furthermore, terminal deoxynucleotidyl transferase-mediated deoxyuridine triphosphate nick-end labeling (TUNEL)-positive apoptosis was markedly increased in the liver of PLEKHO2−/− mice after TNFα/d-GalN (Fig. 5E). These data provide very strong evidence for a critical physiological role of PLEKHO2 in regulating the potentially toxic effects of TNFα. In conjunction with the earlier cell-based approaches, these in vivo findings provide strong support for PLEKHO2 acting as a novel factor in TNFα signaling.

## Discussion

Understanding the molecular mechanism that regulates the ubiquitylation and activation of RIPK1 has been a critical goal of TNFα research. The K63-ubiquitylation of RIPK1, especially on K376, is considered to be critical for preventing its activation. Mice expressing RIPK1^K376R^ which is defective in RIPK1 ubiquitination die during embryogenesis owning to RIPK1 kinase-dependent cell death [15, 16]. In this study, we found PLEKHO2 inhibition of apoptosis and necroptosis is RIPK1 kinase-dependent as well. PLEKHO2 is a protector of RIPK1 ubiquitination and therefore inhibits its activation, subsequently impedes complex II formation and cell death in response to TNFα. However, we did not clarify the way of PLEKHO2 to regulate RIPK1 ubiquitylation. Whether PLEKHO2 facilitates RIPK1 ubiquitination or prevents its de-ubiquitination, especially on K376, needs further comprehensive investigation. In addition, the RIPK1 kinase inhibitor could be used in the mice hepatoxicity model in response to TNFα to prove the role of PLEKHO2 in inhibiting RIPK1 activation in vivo.

We also found PLEKHO2 deficiency results in impaired NF-κB activation after TNFα treatment. Although NF-κB signaling prevents TNFα induced apoptosis, PLEKHO2 may not regulate cell death through NF-κB signaling. Because when treating with TC which blocked NF-κB dependent gene expression, PLEKHO2−/− cells were still more sensitive to the stimuli. As RIPK1 ubiquitination is essential for TNFα induced NF-κB activation, so the impaired NF-kB activation in PLEKHO2−/− cells may be a consequence but not the reason for the regulation of apoptosis.

Our previous studies have reported that PLEKHO1 (also known as CKIP-1), another family member of PH-domain-containing family O, regulates ubiquitination of Smurf1 [17] and TRAF6 [18]. The binding of PLEKHO1 to Smurf1 promotes Smurf1 auto-ubiquitination and E3 ligase activity. In contrast, PLEKHO1 binds TRAF6 but inhibits its auto-ubiquitination and E3 ligase activity. In conjunction with the function of PLEKHO2 reported in this study, these results collectively suggest a common function of PLEKHO family members in ubiquitination regulation. Interestingly, these two members have adverse functions in cell death regulation. In contrast to PLEKHO2, PLEKHO1 promotes apoptosis [19, 20] and inhibits cell proliferation [18, 21]. So the interaction and regulation relationship of these two members should be investigated in future studies.

## Materials and methods

### Mice

Mice were housed in a specific pathogen-free facility and all experimental procedures in mice were approved by the Laboratory Animal Center of the Chinese Academy of Military Medical Sciences and complied with all relevant ethical regulations. For TNFα-induced hepatitis, 8–10 weeks old male PLEKHO2−/− or control C57BL/6 mice were injected intraperitoneally with TNFα (2.5 ng/g body weight) plus d-galactosamine (0.8 mg/g body weight). Mouse survival was monitored over a time period of 36 h. In some experiments mice were killed 6 h after injection, liver samples collected and processed for immunohistochemical analysis, and serum samples were assayed for levels of ALT and AST. The mice numbers were according to the previous study [22].

### Cell culture and reagents

HEK293T cells were cultured in Dulbecco’s modified Eagle’s medium supplemented with 10% (v/v) fetal bovine serum (FBS), 100 mg/ml penicillin and 100 mg/ml streptomycin, and been recently tested mycoplasma contamination by our research group. For the preparation of primary MEFs, PLEKHO2 ± mice were bred to generate PLEKHO2 + / + and PLEKHO2−/− embryos. Embryos were dissected from pregnant females at day 13 post-coitum. MEFs were isolated by standard procedure and cultured in DMEM supplemented with 10% (v/v) FBS. For mouse BMDMs, the femurs and tibias were harvested from mice, washed with PBS, and bone marrow cells were flushed with PBS. The cell suspension was filtered through a 70-μm cell strainer to remove any cell clumps or debris. The single-cell suspension was then cultured in DMEM medium containing 10% (v/v) fetal calf serum (FCS), 30% L929-conditioned media (LCM) used as M-CSF source. To fully differentiate BMDMs, the cells were cultured for 7 days, with fresh media changes every other day.

Nec-1, d-galactosamine, Smac mimetic, and CHX were purchased from Sigma. zVAD was purchased from Bachem, Flag-TNFα was purchased from Enzo Life Sciences, and TNFα was obtained from R&D Systems.

### Immunoprecipitation and Immunoblot

Immunoprecipitation (IP), Immunoblot (IB), and ubiquitination assays were carried out as previously described. Briefly, cells were harvested in the TNE lysis buffer containing 50 mM Tris (pH 7.4), 150 mM NaCl, 1 mM EDTA, 1% NP-40, and a cocktail of protease and phosphatase inhibitors. To remove debris, lysates were centrifuged at 10,000 × *g* for 10 min. Then a two-step IP protocol with 3-h primary antibody incubation and subsequent overnight incubation with ProteinA/G Sepharose beads. Beads were then washed with TNE buffer three times and the bound proteins were analyzed by SDS-PAGE and IB analysis. For ubiquitination assay, a twice IP was performed. The first IP performed as above, then the beads were boiled in 50 μL 1% SDS TNE buffer. Next, the samples were diluted with 500 μL TNE buffer and the second IP was performed. The following antibodies were used: murine caspase-8 (Cell Signaling Technology, 4927), cleaved murine caspase-8 (Cell Signaling Technology, 8592), cleaved caspase-3 (Cell Signaling Technology, 9664), caspase-3 (Cell Signaling Technology, 9665), cleaved PARP (Cell Signaling Technology, 5625), PARP (Cell Signaling Technology, 9542), FLIP_S/L_ (Santa Cruz Biotech,sc-5276), FADD (Enzo Life Sciences, 1F7), RIPK1 (BD, 610459), p-RIKP1 (Cell Signaling Technology, 31122), IKKα (Cell Signaling Technology, 2682), p-IKKα/β (Cell Signaling Technology, 2697), IκB-α(Santa Cruz Biotech, sc-371), p-IκB-α (Cell Signaling Technology, 9246 S), p-p65 (Cell Signaling Technology, 3033 S), p65 (Cell Signaling Technology, 4764 S), p-JNK (Cell Signaling Technology, 4668), p-p38 (Cell Signaling Technology, 4511), HA (MBL, M180-3), Flag (MBL, M185-3L), Myc (MBL, M192-3), PLEKHO2 (Santa Cruz Biotech, sc-165258), and GAPDH (Santa Cruz Biotech, sc-32233).

### Flow cytometry

For flow cytometry analyses of apoptosis, cells were treated with various drugs as indicated. Cells were collected, washed with PBS twice, and stained using the annexin V-FITC/PI according to the manufacturer’s instructions. Stained cells were analyzed with a BD FACSAria III flow cytometer and data were processed using FlowJo software.

### Cell viability assay

Cell viability was assessed by CellTiter-Glo assay (Promega). Cells were seeded at 5 × 10^3^ in black-walled 96-well plates. The following day, media were aspirated and replaced with 200 μL of growth medium containing the relevant concentration of the drug. Once experimental parameters were complete, media was removed from the well, and 100 μL of CellTiter-Glo reagent was added to the samples. The plates were incubated at room temperature for 10 min with gentle agitation and protected from ambient light. Luminescence was then read on a reader (TECAN).

### Quantitative PCR analysis

Total RNA was isolated with TIANGEN (RNAprep pure Cell/Bacteria Kit) and reverse transcription was performed with ReverTra Ace (Toyobo) according to the manufacturer’s instructions. cDNA fragments were amplified by Realtime PCR Master Mix (Toyobo); fluorescence for each gene was detected on an Agilent Mx3005P qPCR System (Agilent, USA). Target gene expression levels were normalized to the housekeeping gene, β-actin, and fold change was calculated using the 2^−ΔΔCT^ method. The primers used for real-time RT-PCR were as following: c-flip, forward 5′-GCTCCAGAATGGGCGAAGTAA -3′ and reverse 5′-ACGGATGTGCGGAGGTAAAAA-3′; caip1, forward 5′-CGCAGCCCGTATTAGAAC-3′ and reverse 5′-AGATTCCCAGCACCTCAG-3′; caip2, forward 5′-CAGAGCACCGCAGACATT-3′ and reverse 5′-GTCCTCAATCGAGCAGAGTG-3′; iκb, forward 5′-GATCCGCCAGGTGAAGGG-3′ and reverse 5′-GCAATTTCTGGCTGGTTGG-3′; a20, forward 5′-CTCAGAACCAGAGATTCCATGAAG-3′ and reverse 5′-CCTGTGTAGTTCGAGGCATGT-3′ and β-actin, forward 5′-TTGTTAC CAACTGGGACG-3′ and reverse 5′-CCAGAGGCATACAGGGAC-3′.

### Immunohistochemical analysis

Liver tissues were fixed in neutral buffered formalin. Following fixation tissues were paraffin-embedded, sectioned at 5 mm, and stained with hematoxylin and eosin (H&E). For TUNEL staining, tissues were stained using the DeadEnd^TM^ Fluorometric TUNEL System (Promega) according to the manufacturer’s instructions. The tissues were examined blinded to the genotype of mice.

### Statistics

Mice with the poor physical condition were excluded before grouping. We used the random number method for random allocation. All values are reported as mean ± SE. Parametric or nonparametric testing was performed as indicated. Kaplan–Meier survival curves were assessed by a log-rank Mantel–Cox test. Student’s *t*-test was used for all other analyses when two groups were compared at one-time point. *P* < 0.05 was used as the cutoff point for significance; *P < 0.05, **P < 0.01, and ***P < 0.001.

## Supplementary information

Figure S1

Figure S1 legend
