# Supplementary figures and images for: PLEKHO2 inhibits TNFα-induced cell death by suppressing RIPK1 activation
Source: Cell Death Dis. 2021 Jul 16;12(8):714. doi: 10.1038/s41419-021-04001-2 (PMC8285381; doi:10.1038/s41419-021-04001-2)

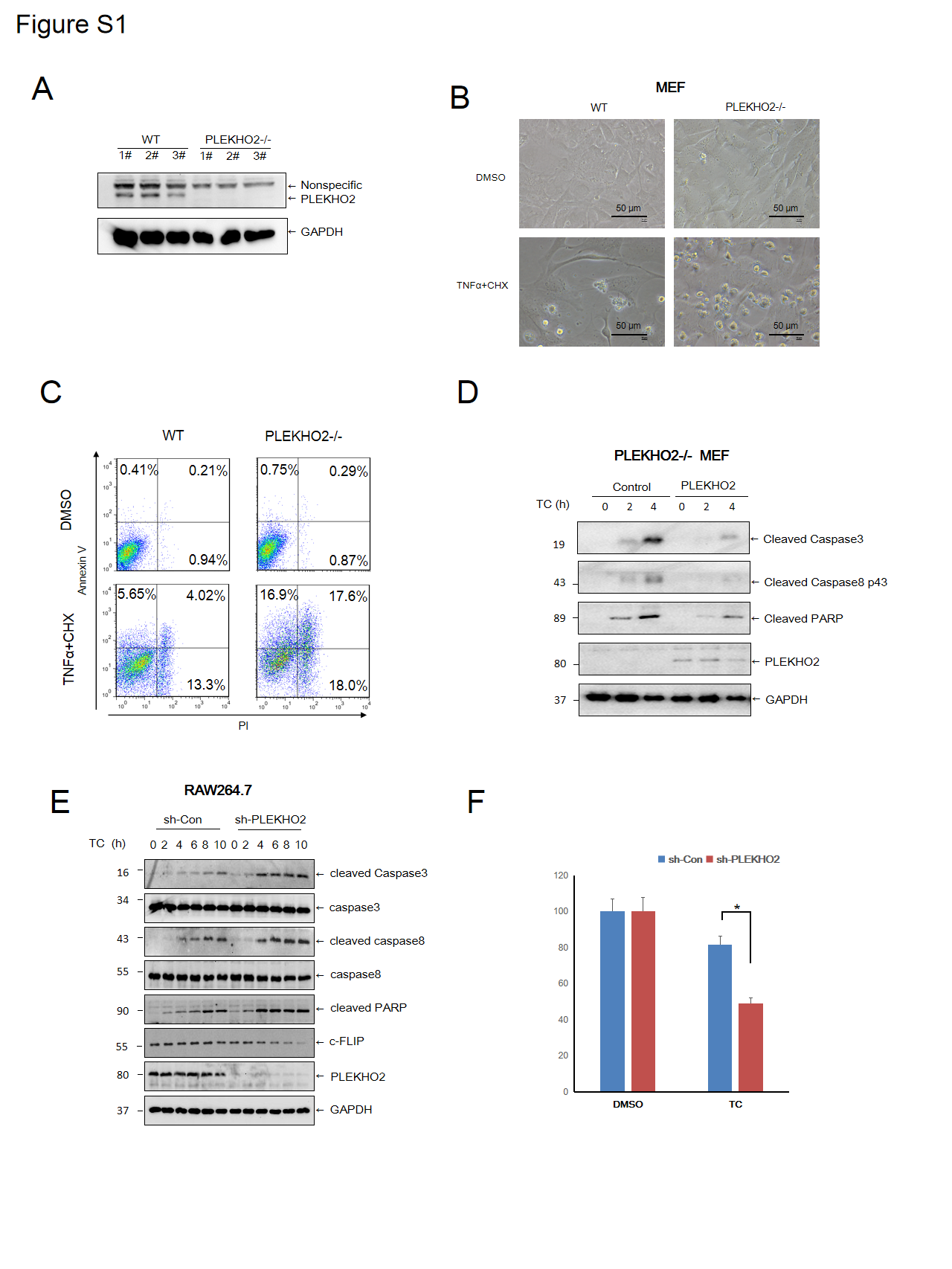

Supplement: Supplementary file 1 — Figure S1 [file 41419_2021_4001_MOESM1_ESM.tif]
