# Supplementary material for: PLEKHO2 inhibits TNFα-induced cell death by suppressing RIPK1 activation
Source: Cell Death Dis. 2021 Jul 16;12(8):714. doi: 10.1038/s41419-021-04001-2 (PMC8285381; doi:10.1038/s41419-021-04001-2)
Supplement: Supplementary file 2 — Figure S1 legend [file 41419_2021_4001_MOESM2_ESM.docx]

**Supplementary figure legends**

**Figure S1 PLEKHO2 inhibits TNFα-induced apoptosis.**

A WT and PLKEHO2-/- MEFs were used for WB to probe PLEKHO2.

B WT and PLKEHO2-/- MEFs were treated with DMSO or murine TNFα (20 ng/mL) and cycloheximide (CHX) (10 μg/mL) for 6 h. Cells were photographed using a phase contrast microscope (bar 50 μm).

C WT and PLKEHO2-/- BMDMs were treated with DMSO or TNFα plus CHX (TC) as B for 8 h and analyzed by flow cytometry for cells staining positive for annexin V and Propidium Iodide (PI) staining.

D Control and PLEKHO2 re-expressed PLKEHO2-/- MEFs were treated with TNFα (20 ng/mL) and cycloheximide (CHX) (10 μg/mL) for indicated periods of time. Cell lysates were probed with indicated antibodies.

E Control and sh-PLKEHO2 RAW264.7 cells were treated with TNFα (20 ng/mL) and cycloheximide (CHX) (10 μg/mL) for indicated periods of time. Cell lysates were probed with indicated antibodies.

F Control and sh-PLKEHO2 RAW264.7 cells were treated with TC (TNFα 20 ng/mL plus CHX 10 μg/mL) for 8 h, cell viability was measured by ATP level.

Results are representative of at least three independent experiments.
